# Introduction. Sensory learning: from neural mechanisms to rehabilitation

**DOI:** 10.1098/rstb.2008.0274

**Published:** 2008-10-31

**Authors:** Paul V. McGraw, Ben S. Webb, David R. Moore

**Affiliations:** 1 Visual Neuroscience Group, School of Psychology, University of NottinghamNottingham NG7 2RD, UK; 2 MRC Institute of Hearing ResearchUniversity Park, Nottingham NG7 2RD, UK

The last decade has seen a spectacular resurgence of scientific interest and advances in our understanding in both the basic neural mechanisms and applications of sensory learning. Given the diverse nature of this problem and the proliferation of data relating to it, we have now reached a critical point where drawing together the various strands of investigation would be extremely beneficial. Different levels of investigation have the potential to inform each other and create situations where step changes in understanding can be made. Detailed knowledge of how sensory learning changes the neurochemistry of the brain is likely to suggest novel pharmacological and behavioural interventions for a range of neurological deficits. Conversely, the success of therapeutic interventions, or lack of it, will provide crucial information on the nature and characteristics of the neural mechanisms underlying the learning process.

This issue draws together researchers working independently across the scientific spectrum—from basic molecular mechanisms through to therapeutic interventions—to communicate their work on sensory learning and neural plasticity within a common forum. The collection of papers, therefore, represents a major interdisciplinary and cross-sensory dissemination of the latest science in this area. The papers have been grouped around four overarching questions. How do we optimize learning? What happens to cortical circuitry during learning? What genes and signalling pathways are involved in learning? How can learning be harnessed to improve the lives of people? It is our hope that this collection of papers, each of which deals with a rapidly evolving aspect of the field, will be of considerable interest to basic scientists and clinicians alike and act as a springboard for future studies.

## 1. A brief historical perspective

Sensory learning, a profound and pervasive aspect of human brain function, has been the subject of philosophical debate and scientific investigation for many centuries. While learning has a long and distinguished history in philosophy, its role in sensory processing was first brought to prominence in the seventeenth century during the great nativism–empiricism debate—a major controversy surrounding the origins of knowledge. In its simplest and most literal form, the nativist position advocated the congenital or innate acquisition of dispositions, related to specific aspects of functioning. Empiricists, on the other hand, proposed that organisms start life with a tabula rasa or blank slate, acquiring knowledge through sensory experience alone. Neither viewpoint was without its problems. The notion that the emergence of specific aspects of function was tied to a discrete and somewhat arbitrary point in time (birth), with little room for acquired characteristics shaped by experience, made many commentators uncomfortable (see the appendix to vol. III of Helmholtz's *Treatise on physiological optics* by J. v. Kries). Similarly, the empiricist thesis, although dominant at the time, faced the problem that sensory data—the substratum of experience—were thought to be accumulated in small isolated and unrelated fragments. There was at the time no obvious way to see how these ostensibly independent pieces of information were combined to mediate the acquisition of perceptual experience. This particular hurdle served as a potent catalyst for the subsequent development of the laws of association (Thorndike 1970), which allowed a link to be made between incoming sensory data and perception.

In the late nineteenth century, the debate again assumed scientific precedence, rebranded as the ‘nature versus nurture’ question. The direction of this debate was further influenced by two contributing factors. The first was the influence of the distinguished German physicist Hermann von Helmholtz who put forward his seminal ideas on ‘unconscious inference’. Helmholtz asserted that sensory perception contained an element of inference and that this process was derived unconsciously and in its entirety from the accumulation of past experience (Helmholtz 1910). With regard to patterns of retinal stimulation, he commented:

Inasmuch as in an overwhelming majority of cases, whenever the parts of the retina in the outer corner of the eye are stimulated it has been found to be due to external light coming into the eye from the direction of the bridge of the nose, the inference we make is that it is so in every new case whenever this part of the retina is stimulated; just as we assert that every single individual now living will die, because all previous experience has shown that all men who were formerly alive have died.

(Helmholtz 1910, pp. 4–5)

The notion of unconscious inference therefore ascribed a direct role to learning in shaping sensory perception. The second factor was the emergence of Darwinian biology (Darwin 1859). Although primarily concerned with natural selection and the adaptation of species from one generation to the next, its central tenet was adaptation to the environment. If it were possible to shape the innate properties of an organism from birth, in a way that bestowed some functional advantage that could be carried to the next generation, the logical conclusion was that this must surely be based on how the organism interacted with its environment. This then presented an opportunity for achieving a middle ground between the extremes of nativism and empiricism. In the light of new evolutionary theory, the most important challenge in human sensory science became understanding what functions and capacities of perceptual processing are present at birth and how these are subsequently influenced by sensory experience acquired through interacting with our physical environment.

## 2. What is sensory learning?

Learning itself takes many forms. This issue is concerned with sensory or perceptual learning and the neural mechanisms that underpin this process. A useful functional definition of sensory learning was provided by Gibson (1969) in her influential textbook on the subject. She stated:

Perceptual learning then refers to an increase in the ability to extract information from the environment, as a result of experience and practice with stimulation coming from it. That the change should be in the direction of getting better information is a reasonable expectation, since man has evolved in the world and constantly interacts with it. Adaptive modification of perception should result in better correlation with events and objects that are sources of stimulation as well as an increase in the capacity to utilize potential stimulation.

(Gibson 1969, pp. 3–4)

We are probably all familiar with the different examples of sensory expertise that Gibson alludes to in her book: the lore of a wine connoisseur that can discriminate subtle differences in grape varietals; the musician's ear that can discriminate fine changes in the temporal structure of a musical piece; the experienced eye of a radiologist that can detect almost imperceptible shadows in an X-ray image; and the remarkable ability of a blind person to echo-locate objects and avoid them by repeatedly tapping a cane. The important point to note here is that, in each of the above examples, the physical information available to our senses is identical, yet our powers of discrimination or detection can be very different.

This raises a number of important questions. First, what are the upper limits to these types of sensory enhancement and what aspects of sensory processing set the constraints? Second, are the benefits specific to the type of task and stimulus set or do they generalize to other situations? Finally, how permanent are the effects: if exposure to the sensory information is reduced or eliminated, do the previously learned benefits endure? Each of these issues has profound implications for the neural mechanisms mediating the learning process and is addressed in the first section of this issue. The first three papers could be considered as the backbone of this issue, providing perspectives, recent data and models drawn from specialists in visual and auditory learning (Ahissar *et al.* 2009; Fahle 2009; Wright & Zhang 2009).

Taking the visual system as an example, it has been widely documented that practice improves performance on a multitude of tasks (for reviews see Gilbert *et al.* 2001; Fine & Jacobs 2002). When making challenging sensory judgements, improvements in perceptual performance are tightly coupled to the particular task and stimulus arrangement used during the initial training period. For example, training on a position discrimination task produces performance improvements that are tightly coupled to the trained retinal location (Fahle *et al.* 1995), but vanish when the same stimulus is presented at a new location in space. Similar selectivity has also been found for many other visual discriminations, including orientation and spatial frequency (Fiorentini & Berardi 1980; Karni & Sagi 1991).

The relevance of this type of specificity is the implication that sensory learning might be mediated by early visual cortex where cells have small receptive fields and are selective for the same image properties (e.g. orientation, spatial frequency and position). However, learning of more complex image structures, the encoding of which requires larger and more sophisticated receptive fields, typical of higher cortical levels in the processing hierarchy (Sakai & Miyashita 1994; Zohary *et al.* 1994), does not show the same level of specificity. The challenge of reconciling the degrees of specificity and generalization of sensory learning is taken up in this first section (Ahissar *et al.* 2009; Fahle 2009; Wright & Zhang 2009). The answer may lie in the fact that learning itself is implemented at multiple levels of cortical analysis (Ahissar *et al.* 2009) and flexibly updates downstream sensorimotor representations (Fahle 2009). In addition, new data from the auditory domain are presented that reveal a wide variety of generalization patterns across a number of basic auditory tasks (Wright & Zhang 2009). Understanding, extending and exploiting this latter aspect of learning is key to the development of sensory learning as a therapeutic tool.

In the second set of papers, the authors ask how different networks, at the level of sensory systems, contribute to learning. The challenge here is to understand how populations of neurons, either within a single sensory system (Hoffman & Logothetis 2009) or across different sensory systems (King 2009), cooperate to produce the perceptual benefits of learning. A good example of the former can be found in the field of object recognition, where a rich three-dimensional representation of a complex object must be constructed from the sparse two-dimensional pattern of light first imaged on the retina. What is the nature of this constructive process and what role does experience play in it? Related to this problem is the fact that when we change our position with respect to an object, our retinal representation is altered substantially. In spite of this, we are still able to identify this new image as an altered representation of the same object. Clearly, in order to represent objects in this viewpoint-invariant fashion, the brain needs to employ an active process that is heavily reliant on previous experience. Helmholtz sums up this problem succinctly:

If the objects had simply been passed in review before our eyes by some foreign force without our being able to do anything about them, probably we should never have found our way amidst such an optical phantasmagoria; any more than mankind could interpret the apparent motions of the planets in the firmament before the laws of perspective vision could be applied to them.

(Helmholtz 1910, p. 31)

The critical point here is the role of experience: we need to learn that if we vary the conditions under which an object is viewed, by, let us say, changing our position, the altered retinal representation is a direct result of these actions and not a change in the structure of the object itself. In the fourth paper in this issue (Hoffman & Logothetis 2009), the authors investigate how we learn to identify new objects and object categories and reveal the brain areas that are critical to the learning process.

It is certainly true to say that learning about objects often involves more than a single sense. This is readily evident when a young child explores a new object for the first time. Repeated viewing of the object from different angles is combined with coordinated tactile exploration using the hands (and often the mouth). In this example, experience is being used to build up congruent associations between visual and tactile stimulation. Similarly, when we are required to localize an object in external space, associated visual, auditory and tactile cues need to be combined in order to form coherent spatial maps. Although each system is capable of providing an estimate of object location, the superior sensitivity of the visual system for judgements of this type means that, during development, auditory and tactile spatial maps are calibrated relative to visual input. However, new evidence suggests that the strategy the brain uses to flexibly integrate multisensory cues changes later in childhood (Gori *et al.* 2008). Rather than a single modality, such as vision, dominating the coordination of sensory input, combinations of inputs across the senses occur in a way that enhances the localization and discrimination of sensory stimuli. The fifth paper (King 2009) in this issue examines the role of early multisensory experience in establishing coordinated maps of visual and auditory space and details modifications to the neural circuits underpinning spatial hearing that can be induced with and without the influence of the visual system.

## 3. What are the cellular and molecular neurodynamics of learning?

Returning to our earlier example of the wine connoisseur, it is reasonable to assume that, at birth, we are all endowed with the same neural hardware. Given this fact, it is important to understand what experience-dependent changes to the functional properties of brain circuits and central structures enable changes in perceptual ability. The wine expert illustrates that sensory learning can and does occur later in adulthood: indeed, learning is generally viewed as a lifelong process. Yet it is just after birth, during a ‘sensitive period’ of development, that experience-dependent changes are most pronounced. Although we are born with rudimentary cortical circuitry in place, normal development requires rich sensory experiences. Classically, the role of sensory experience has been studied by manipulating sensory input and charting the consequent structural and functional reorganization of connections. This approach has been indispensable in revealing the neural mechanisms that allow us to adapt to new or altered sensory input. A more comprehensive understanding of the neurodynamics of the nervous system is essential to understanding why and how some functions deviate from the developmental plan in certain disorders, and is critical for developing novel rehabilitative strategies. It is also possible that many of the mechanisms that drive experience-dependent changes to cortical function during development may display important commonalities with those that drive sensory learning later in life. At present, we do not know if this is the case. The three papers that comprise the next section of this issue examine the anatomical pathways and cellular and molecular mechanisms responsible for precipitating experience-dependent changes in the sensory cortex. The authors present information gathered from visual (Smith *et al.* 2009; Tropea *et al.* 2008) and somatosensory (Fox 2009) cortex.

The fact that the cortical circuitry of the brain can be altered by sensory experience has been known for quite some time. The term ‘plasticity’ is used to describe this process and was originally applied to the enduring alterations in connection weights that occur when activity in an input element is correlated with the rise in activity of a receptor element (Konorski 1948). This system of strengthening synaptic connections (known as potentiation) based on sensory experience offered a new framework for understanding the behavioural changes associated with learning (Hebb 1949). However, the theoretical and computational limitations of early Hebbian learning have hastened the development of alternative models to explain long-term and homeostatic synaptic plasticity. The major change has been to accommodate a role for synaptic depression, or the weakening of synaptic connections between neurons that are not sufficiently co-active. The empirical analogues of synaptic potentiation and depression are long-term potentiation (LTP) and long-term depression (LTD). Following in the footsteps of archetypal Hebbian learning, a number of models have evolved based on the rate of pre- and post-synaptic neural firing (rate-based models), or differences in timing between pre- and post-synaptic firing (timing-based models). Perhaps one of the most influential exemplar of the former class is the BCM model (Bienenstock *et al.* 1982), which incorporates a ‘sliding’ modification threshold, operating between LTP and LTD. A range of biophysical models have also been developed, most of which are based on the role of calcium gradients in the induction of synaptic plasticity (Gamble & Koch 1987; Yang *et al.* 1999).

Bidirectional synaptic plasticity resulting from modification of visual input (lid suture) can be considered in three stages. First, LTD weakens the response from the deprived eye. Following this, prolonged deprivation produces changes in the modification threshold. This, in turn, supports potentiation of responses, via LTP, of the open eye. The first step in this three-stage model has been widely investigated, but, here, work is presented that examines in detail the neural mechanisms that govern the later two stages (Smith *et al.* 2009). The next paper examines the roles of candidate molecules and mechanisms in mediating activity-dependent changes in ocular dominance column plasticity in the visual cortex (Tropea *et al.* 2008). The authors identify a surprisingly large range of molecules, each of which contributes to either feed-forward (changes specific to the eye with altered input) or feedback (cell-wide changes to synapses of both eyes) mechanisms of plasticity—processes that ultimately combine to produce binocular competition. These studies draw on a range of enabling technologies such as *in vivo* visualization of structural dynamics via high-resolution imaging with fluorescent activity probes, the use of genetically modified mice and microassay screens to identify the genetic signalling pathways that mediate plasticity. In the final paper in this section (Fox 2009), the anatomical pathways, mechanisms of synaptic and structural plasticity and the role of gene expression in plasticity and cortical stability are detailed for the somatosensory cortex. This work forms an essential cornerstone to understanding and enhancing functional rehabilitation after stroke.

## 4. Can sensory learning be harnessed as a therapeutic tool?

In the final section of this issue, a somewhat broader perspective is taken. The authors consider ways in which science-based sensory learning can be implemented within a therapeutic framework to ameliorate a number of common neurological conditions. Examples from both the visual and auditory domains are considered. The first two papers (Levi & Li 2009; Mitchell & Sengpiel 2009) deal with one of the most common causes of abnormal sensory development in the visual system, namely amblyopia or ‘lazy eye’. This condition is found in approximately 3–4% of the population and is responsible for the vast majority of children's hospital eye appointments in the UK. The condition is associated with the presence of some obstacle to normal sensory development during the sensitive period. This commonly takes the form of unequal refractive errors between the two eyes (anisometropia), misalignment of the visual axes (strabismus) or, more rarely, visual deprivation (e.g. congenital cataract). The traditional treatment for this condition is occlusion of the non-deprived eye (patching) for long periods—a treatment method that is unpopular with both children and parents. More importantly, occlusion therapy produces little or no visual benefit in approximately one-third of all cases and is rarely undertaken in older children (beyond 8–9 years) due to poor success rates. These factors have prompted scientists working in this area to search for viable treatments that could either augment or, possibly, supplant standard treatment protocols.

The first paper in this final section (Mitchell & Sengpiel 2009) examines ways in which occlusion therapy can be enhanced. It was previously thought that aggressive patching regimens, involving almost continual occlusion of the non-deprived eye, were most effective. However, more recent animal and clinical studies have shown that part-time occlusion therapy can be just as effective as full-time treatment without any of the unwanted side effects (e.g. reverse deprivation). Although now widely accepted, the reasons for this have remained largely unknown. A series of studies conducted on visually deprived animals reveal the critical role of concordant binocular input. These findings suggest that traditional occlusion therapy could be significantly enhanced by the provision of appropriate periods of binocular exposure.

More recently, the role of sensory learning in enhancing visual performance in humans with amblyopia has come to the fore and is covered in the next paper (Levi & Li 2009). Several studies have now shown that both children and adults with this condition can improve their performance via extensive practice on a challenging visual task (Levi 2005; Li *et al.* 2005). The importance of this work is threefold: first, the improvements generated on one task appear to transfer to another; second, the visual benefits can be realized over much shorter time scales than traditional therapy; and, third, it offers the first treatment opportunity for adults with amblyopia.

In the final paper (Moore *et al.* 2009), the authors show that the same learning-based approach can be adopted in the treatment of listening and language problems. By comparison, the results to date are even more spectacular than those obtained in the visual domain. However, applications of auditory learning in children have yielded highly variable results and have highlighted the need for rigorous experimental design including appropriate controls. Here, the authors outline strategies for promoting the persistence of learning and maximizing the transfer of learning effects between tasks. They also offer clues as to why some studies have produced such apparently contradictory results and offer interpretations of the likely sources of the extremely transferable learning observed.

The clinical results obtained so far are extremely encouraging and build nicely on the advances made in basic science. Having said this, none of these learning-based training procedures have yet been the subject of a randomized controlled trial. This will be essential in the near future and will provide a much more robust and reliable indication of their efficacy. This collection of papers has identified a number of fundamental challenges that will need to be addressed. At first glance, these seem every bit as daunting as those faced by previous generations of scientists. But given the rapid development of enabling technologies and a willingness to embrace a mutually informed multidisciplinary approach, there are good reasons to believe that a fuller understanding of the mechanisms of neural plasticity and how to exploit them is within reach.
